# Geostatistical evaluation of integrated marsh management impact on mosquito vectors using before-after-control-impact (BACI) design

**DOI:** 10.1186/1476-072X-8-35

**Published:** 2009-06-23

**Authors:** Ilia Rochlin, Tom Iwanejko, Mary E Dempsey, Dominick V Ninivaggi

**Affiliations:** 1 Division of Vector Control, Suffolk County Department of Public Works, 335 Yaphank Avenue, Yaphank, New York 11980-9744, USA; 2 Suffolk County Department of Environment and Energy, 335 Yaphank Avenue, Yaphank, New York 11980-9744, USA

## Abstract

**Background:**

In many parts of the world, salt marshes play a key ecological role as the interface between the marine and the terrestrial environments. Salt marshes are also exceedingly important for public health as larval habitat for mosquitoes that are vectors of disease and significant biting pests. Although grid ditching and pesticides have been effective in salt marsh mosquito control, marsh degradation and other environmental considerations compel a different approach. Targeted habitat modification and biological control methods known as Open Marsh Water Management (OMWM) had been proposed as a viable alternative to marsh-wide physical alterations and chemical control. However, traditional larval sampling techniques may not adequately assess the impacts of marsh management on mosquito larvae. To assess the effectiveness of integrated OMWM and marsh restoration techniques for mosquito control, we analyzed the results of a 5-year OMWM/marsh restoration project to determine changes in mosquito larval production using GIS and geostatistical methods.

**Methods:**

The following parameters were evaluated using "Before-After-Control-Impact" (BACI) design: frequency and geographic extent of larval production, intensity of larval production, changes in larval habitat, and number of larvicide applications. The analyses were performed using Moran's I, Getis-Ord, and Spatial Scan statistics on aggregated before and after data as well as data collected over time. This allowed comparison of control and treatment areas to identify changes attributable to the OMWM/marsh restoration modifications.

**Results:**

The frequency of finding mosquito larvae in the treatment areas was reduced by 70% resulting in a loss of spatial larval clusters compared to those found in the control areas. This effect was observed directly following OMWM treatment and remained significant throughout the study period. The greatly reduced frequency of finding larvae in the treatment areas led to a significant decrease (~44%) in the number of times when the larviciding threshold was reached. This reduction, in turn, resulted in a significant decrease (~74%) in the number of larvicide applications in the treatment areas post-project. The remaining larval habitat in the treatment areas had a different geographic distribution and was largely confined to the restored marsh surface (i.e. filled-in mosquito ditches); however only ~21% of the restored marsh surface supported mosquito production.

**Conclusion:**

The geostatistical analysis showed that OMWM demonstrated considerable potential for effective mosquito control and compatibility with other natural resource management goals such as restoration, wildlife habitat enhancement, and invasive species abatement. GPS and GIS tools are invaluable for large scale project design, data collection, and data analysis, with geostatistical methods serving as an alternative or a supplement to the conventional inference statistics in evaluating the project outcome.

## Background

The salt marsh is a globally important ecosystem in high to middle latitudes along the coastline [1]. Ecologically, salt marshes provide a nutrient rich interface between terrestrial and marine environments utilized by a great variety of animal species. The salt marsh habitat is also of a significant public health importance due to mosquito vector species that have adapted to this harsh environment. Pathogens transmitted by salt marsh mosquitoes include the malaria parasite vectored by *Anopheles atroparvus*, *An. sacharovi*, and *An. labranchiae *in Europe and the Middle East [2], Venezuelan Equine Encephalitis virus vectored by *Aedes sollicitans *and *Ae. taeniorhynchus *in the Americas [3], California group encephalitis viruses vectored by *Ae. dorsalis*, *Ae. caspius*, and *Ae. melanimon *in Europe and western North America [4], and Ross River virus vectored by *Ae. camptorhynchus *and *Ae. vigilax *in Australia [5]. Many of the salt marsh *Aedes *mosquitoes are also important biting pests species in coastal population centers and tourist areas [2,5].

The salt marsh mosquito fauna of Long Island, New York is representative of the Atlantic seaboard of the continental US consisting of 4 species, *Ae. sollicitans*, *Ae. cantator*, *Ae. taeniorhynchus*, and *Cx. salinarius *[6,7]. *Aedes sollicitans *is considered the main epidemic vector of Eastern Equine Encephalitis virus (EEEv) in coastal areas of eastern US [8-10]. The virus has been repeatedly isolated from the wild populations of *Ae. sollicitans *in this region [11,12] and occasionally from *Ae. cantator *and *Ae. taeniorhynchus *[11,13]. *Culex salinarius *is a potentially important epidemic vector of EEEv [14] with multiple field isolations of the virus [15-17]. This mosquito species is also one of the main vectors involved in West Nile virus (WNV) human transmission [18-20]. In addition to numerous WNV isolations from field collected *Cx. salinarius*, the virus was detected in *Ae. sollicitans *(including specimens collected on Long Island) and occasionally in *Ae. cantator *and *Ae. taeniorhynchus *[21,22]. All three salt marsh *Aedes *species can potentially transmit WNV to humans [23,24], although only *Ae. sollicitans *has been associated with the risk of WNV transmission to humans [25].

The public health importance of the salt marsh mosquitoes in the coastal areas of eastern US was apparent long before the discovery of the mosquito-borne viruses. Massive infestations by *Ae. sollicitans *and *Ae. taeniorhynchus *led to the establishment of most, if not all, coastal mosquito control districts [26,27]. Abatement of larval mosquitoes on the salt marsh employed both chemical and habitat modification techniques including ditching of the marsh surface to allow rapid draining of small pools harboring mosquito larvae. Similarly to many coastal areas, Long Island salt marshes were grid ditched in the late 1930s [28]. Although reasonably effective as a mosquito control tool, universal grid ditching was perceived as unnecessary in those parts of salt marshes not producing mosquitoes, and unsatisfactory from ecological and resource conservation perspectives [29]. These concerns resulted in the development of an Open Marsh Water Management (OMWM) technique with the dual goal of non-chemical mosquito control and marsh conservation [29-31]. OMWM targets specific areas of known mosquito larval habitat by employing tidal channels, ponds, and shallow radial ditches to remove these habitats [32]. Tidal channels are designed to improve water circulation and to restore natural tidal regime, while small ponds are created to replace clusters of depressions where mosquito larvae proliferate. Shallow radials connect ponds with scattered depressions to allow access by larvivorous fish. With some modifications, OMWM has been put into practice in many mosquito producing salt marshes along the US Atlantic coast from Massachusetts to Florida, California, and Australia (reviewed in [33]). Invariably, satisfactory results were reported, with the initial estimates suggesting elimination of 40–60 billion mosquitoes annually for every 1,000 OMWM acres [30]. Direct measurements of mosquito production, although infrequent, documented significant reductions (> 85%) in larval or adult mosquito levels (reviewed in [33,29]); however, many studies relied on rather qualitative observations such as number of complaints to assess the outcome. A recent OMWM survey for US Fish and Wildlife Service (USFWS) [34] utilized before-after-control-impact (BACI) design [35] allowing statistically rigorous analysis of the technique's effects [36]. Although we adopted a similar BACI approach for our project, the two studies differed significantly in scope, goals, and methodology.

Suffolk County occupies most of Long Island, New York and employs both pesticides and water management to control salt marsh mosquitoes. To investigate alternative approaches in compliance with the pesticide usage reduction goal set by the County government, a partnership with USFWS was initiated for a pilot integrated marsh management project at Wertheim National Wildlife Refuge (Wertheim NWR). The following goals of the project included both marsh restoration and mosquito control components: 1) to decrease mosquito production and, by extension, pesticide usage by utilizing OMWM approach, 2) to reduce the vigor and extent of the invasive reed *Phragmites australis *[37], 3) to naturalize marsh surface by eliminating grid ditching, and 4) to maintain or enhance fish and wildlife habitat. This article describes the planning and implementation of the project and focuses on quantitative outcome evaluation for mosquito control. Geographic information systems (GIS) and GPS technology were systematically employed at each stage of the project allowing efficient use of limited resources and enabling development of novel methodology for assessing the project impacts.

## Methods

### Study area

**Figure 1:**
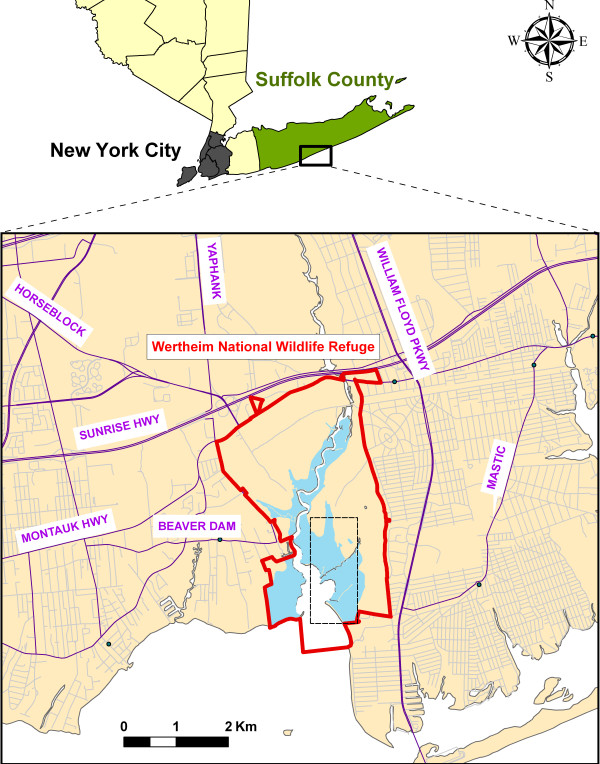
**The Wertheim project study area**. Red line shows Wertheim NWR boundary; dashed line indicates approximate geographic extent of the study area. Tidal wetlands are indicated in blue. Major roads (purple) and residential streets (gray) are also shown.

The project site was located in the ~1,033 ha Wertheim NWR administered by USFWS (Figure 1). Tidal wetlands of Wertheim NWR (~262 ha) and the adjacent areas (~103 ha) form the largest continuous salt marsh on Long Island. The Wertheim NWR salt marshes are comprised of "low" marsh of *Spartina alterniflora *where daily tidal flooding occurs, and "high" marsh with intermittent flooding during storms or high tides occupied by three major plant species – *S. patens*, low-form *S. alterniflora*, and the invasive *Phragmites australis*. These relatively undisturbed and protected areas are surrounded by heavily suburban environment. About 105,000 people (Census 2000) live within 8 km from Wertheim NWR's tidal wetlands, at a typical distance covered by the migratory flight of 50% of newly emerged salt marsh *Aedes *[26]. Thus, public health and quality of life issues associated with salt marsh mosquito species necessitate an active larval control program at Wertheim NWR and adult control program in most of the surrounding communities.

### Project planning

**Figure 2:**
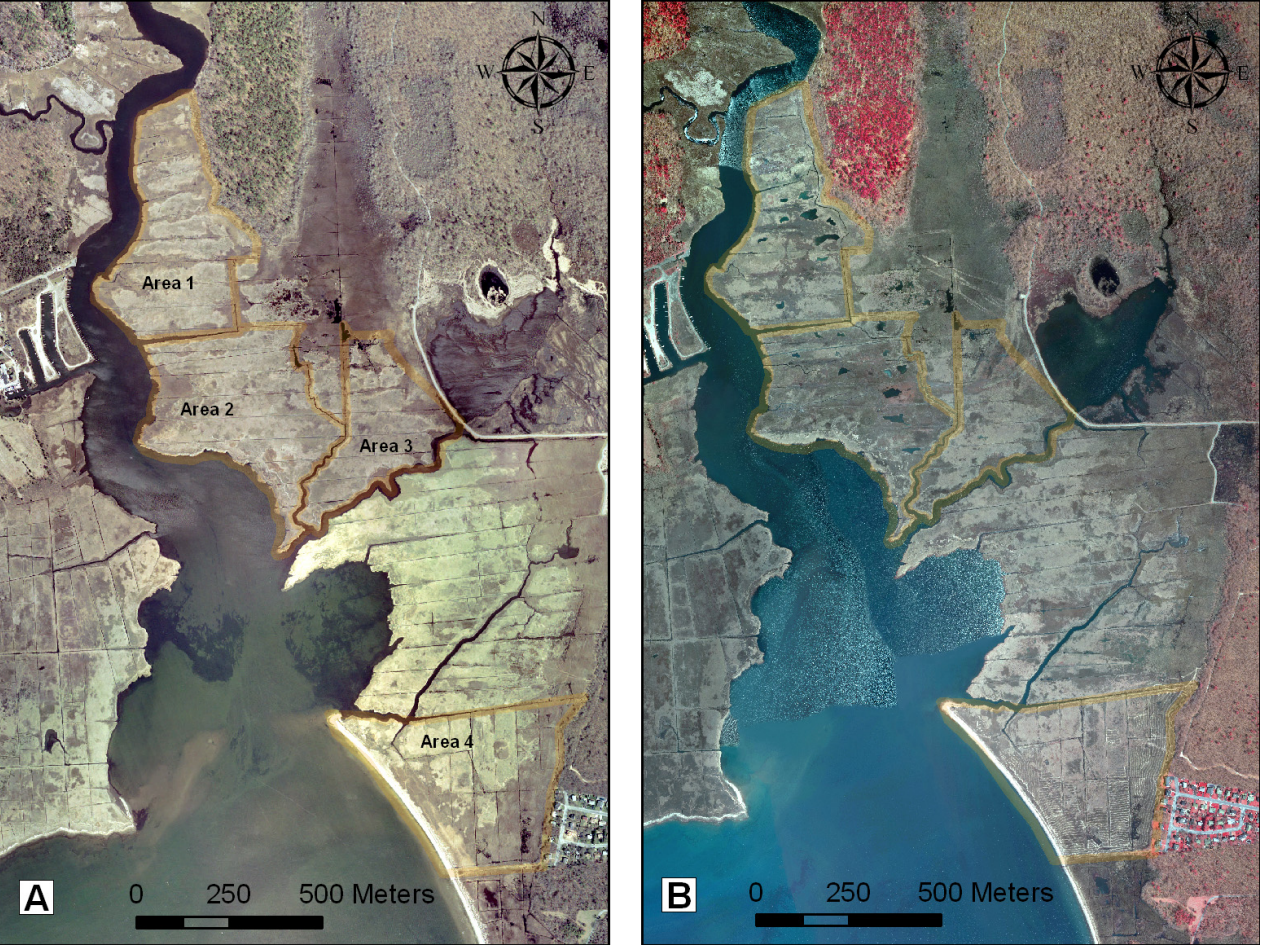
**Aerial view of the study areas in 2004 (A, pre-project) and 2007 (B, post-project)**. Treatment: Areas 1 and 2, Control: Areas 3 and 4.

The conceptual framework of the project was designed to meet the needs of both vector control and natural resources conservation. OMWM was proposed as a sustainable solution for mosquito control, with a potential to enhance fish and wildlife habitat. The two major features of the OMWM component were ponds and their shallow connectors to tidal channels, which were designed to improve pond water quality and to facilitate access by marine species. Marsh surface restoration and invasive species control were aimed at improving the biological functions of the salt marshes. Eliminating grid ditching was the key step in marsh restoration, while new or enhanced tidal channels/creeks were intended to provide better tidal flow in the *P. australis *infested portions of the marshes. In accordance with the paired BACI design of the study, two treatment and two control areas of the marsh were delineated on-screen in ArcGIS environment using aerial ortho-photographs with creeks and ditches served as natural boundaries. Area 1 (16.0 ha) and Area 2 (18.9 ha) were designated as the treatment areas, whereas Area 3 (10.7 ha) and Area 4 (18.5 ha) were designated as the control areas (Figure 2). For preliminary surveillance, loci of mosquito breeding and vegetation cover were georeferenced by GPS hand-held devices and used to create the base map. New hydrologic features (i.e. ponds, tidal channels, and connectors) in the treatment areas were plotted on-screen in ArcGIS environment using the base map. A small number of mosquito ditches suitable for hydrologic connectivity were retained for naturalization; most grid-ditches were targeted for filling in. Following the delineation of the areas and the proposed alterations, all the required permits were obtained from the local, state, and federal agencies.

### Project implementation

To assist in mapping out the proposed hydrologic features on the ground, a network of georeferenced points was established in the treatment areas using Trimble^® ^GPS receivers (Trimble Navigation Ltd., Sunnyvale, CA). The outline of ponds, tidal channels, connectors, and larval habitat targeted for elimination by filling in dense concentrations of larvae producing potholes [29] were staked out using the hydrologic feature map and georeferenced points as a guide. The stake locations were recorded by hand-held GPS receivers and the data were visualized in ArcGIS overlaying the hydrologic feature map. The stake positions were then adjusted if necessary. The following alterations were made in Area 1 (March 2005) and in Area 2 (February-March, 2006). Ponds, tidal channels, and connectors were constructed using low-ground pressure (< 2 psi) machinery. The majority of pre-existing mosquito control ditches in the marsh were filled with the material extracted during the pond excavation process using rotary ditchers and excavators. The remaining ditches were naturalized by adding curves and other features commonly found in natural salt marsh channels and creeks. Additional tidal channels were created with the overarching goal of increased tidal circulation into the interior of the salt marshes. The excess pond/tidal creek excavated material was used to grade hummocky high marsh terrain where larval habitat had been found by the preliminary surveillance (i.e. "backblading").

### Entomological data collection

Two sampling procedures were employed to collect larval data. A systematic sampling protocol [34] was carried out to establish random transects (n = 5 in Areas 1 and 2 each, n = 4 in Areas 3 and 4 each) with sampling stations distributed equally at every 40 meters with sampling taking place at and between the stations (i.e. every 20 meters) resulting in a total of 24 sampling points in each Areas 1 and 2, and a total of 20 sampling points in each Areas 3 and 4. These transects extended from the upland portion of the salt marsh seaward into the low marsh. A second procedure utilized targeted sampling, a more common method used by mosquito control professionals, whereby suitable larval habitats, i.e. pools of standing water, were searched for mosquito larvae across all 4 Areas. These surveys intended to be comprehensive, so the majority of potential larval habitats within an area were sampled each time. At least 25 samples were obtained per area per visit unless the marsh surface was either dry (no standing water) or completely flooded.

Each area was visited weekly for targeted sampling from early May to mid September, a period corresponding to the active mosquito season in this region. Additionally, transect sampling was carried out monthly 4 to 7 days following a high tide inundation to maximize the chances of finding mosquito larvae on the salt marsh [34]. Mosquito larvae were collected using standard mosquito dippers (Bioquip Inc., Rancho Dominguez, CA). Custom database design software for Windows CE (Visual CE, Syware, Cambridge, MA) was developed to run on a hand-held personal digital assistant (PDA; Dell Axim™ X51, Dell Inc., Round Rock, TX) coupled with a GPS device (GPSlim 236, Holux Technology Inc., Hsinchu, Taiwan). The program recorded the following data: geographic coordinates, time, number of mosquito larvae and pupae, type of habitat, sampling method with transect/station information, and comments. The data were uploaded directly into a MS Access database using Microsoft ActiveSync. For data quality assurance, the sampling points were visualized in ArcMap and any errors were identified and corrected.

As a rule, 1 to 5 typical larval samples from each area were brought to laboratory for microscopic species identification using morphological mosquito keys [38]. First instar larvae were allowed to progress to later instars, and larvae of *Culex *spp. were allowed to emerge as adults to confirm positive identification. Adult *Cx. salinarius *were separated from those of *Cx. pipiens *by using morphological characters [38] as well as molecular techniques [39].

### Entomological data evaluation and statistical analysis

Three parameters were evaluated to assess the outcome of the project: frequency and geographic extent of larval production, intensity of larval production, and the overall impact of the project (both OMWM and restoration components) on mosquito larval habitat. In accordance with BACI design, the analyses were performed on aggregated "Before and After" data and also followed through time to compare "Control" and "Impact" (i.e. treatment) areas to identify changes attributable to the intervention.

Presence or absence of mosquito larvae, a dichotomous variable, was used to evaluate geographic patterns of larval production. Each sample was thus classified as either 1 (= positive dip) or 0 (= negative dip). Global and Anselin Local Moran's I were calculated to assess the overall spatial patterns and local geographic clusters of positive or negative dips using ArcMap 9.3 software (ESRI Inc, Redlands, CA). Normalized Z-scores, or the number of standard deviations, were interpreted to represent clusters (Z-score > 2.0), outliers (Z-score < -2.0), or random distribution (-2.0 < Z-score < 2.0) at statistical significance *P *< 0.05. To visualize a statistical surface of mosquito larval production over the entire study area, Voroni tessellation was performed to create Thiessen polygons around each data point classified by presence or absence of mosquito larvae and the Z-score. Adjacent polygons in the same category were dissolved and smoothed to produce the final map.

To define statistical significant clusters of positive and negative dips spatially and temporarily, a spatial scan cluster analysis was carried out using free SaTScan™ software [40]. Positive dips (the case file) and negative dips (the control file) were analyzed by space-time statistic with Bernoulli probability model for dichotomous data employing the following settings: year as the time period, maximum non-overlapping spatial cluster size of 0.5 km radius roughly corresponding to the extent of each treatment and control area, scan for high and low values, and 4-year temporal window with pure spatial clusters (i.e. present each year). The statistical significance was calculated by Monte-Carlo simulation with 9999 replications.

To determine the intensity of mosquito larval production (termed "breeding intensity"), an ordinal scale was developed based on field observations. Dips with 1 to 2 larvae per dip were classified as "low" (rank = 1), those with 3–5 larvae per dip as "intermediate" (rank = 2), and those with > 5 larvae per dip as "high" (rank = 3). Only positive dips were included in the analysis using global (General G) and local (Gi*) Getis-Ord statistic in ArcMap 9.3 to assess the overall distribution of breeding intensity values and to identify geographic locations with elevated breeding intensity, i.e. non-random clusters. Similarly to Moran's I statistic, normalized Z-scores were interpreted to represent clusters (Z-score > 2.0), outliers (Z-score < -2.0), or random distribution (-2.0 < Z-score < 2.0) at statistical significance *P *< 0.05. To visualize distribution of positive dips on the marsh surface, kernel density was calculated using 5 × 5 meter grid and 50 meter search radius.

### Post-project problem area characterization

ArcMap 9.3 buffering tool and sampling tools available with Hawth's ArcMap extension [41] were utilized to evaluate changes in mosquito larval habitat before and after the treatment. Specifically, the impact of filling in mosquito ditches (a marsh restoration technique) on larval habitat was assessed by determining proportion of positive dips at close (< 5 meters), medium (5–15 meters), or long (> 15 meters) distance range. All non-spatial statistical analyses were conducted in SPSS v. 15.0 (SPSS Inc, Chicago, IL) software and assumed statistical significance at *P *< 0.05.

### Mosquito abatement

Mosquito abatement measures consisted of aerial application of larvicides by helicopter. Routine mosquito control program continued throughout the study period using the standard criteria set by the state regulatory agencies and USFWS. At least 25 samples were required from each area to meet the minimum larviciding threshold of 0.2 larvae per dip. Other considerations included the extent of the infested area (i.e. total number of positive dips), weather, and environmental conditions directly affecting mosquito larval habitat such as marsh flooding. These criteria were uniformly applied without regard to the status (impact or control) of the particular location to determine whether a larvicide application was justified. Two types of larvicides were used in the control program. Vectobac 12AS (*Bacillus thuringiensis *var. *israelensis*; ValentBioscience Corp.) is a liquid bacterial product applied when early larval instars (stages 1–2) were detected. Altosid Liquid Larvicide Concentrate (methoprene; Central Life Science/Wellmark™) is an insect growth regulator applied against late instar larvae (stages 3–4). When both early and late larval instars were present simultaneously, a combination of Vectobac and Altosid was used.

Before and after treatment effects on number of larvicide applications and proportion of time the larviciding threshold was reached were analyzed according to the published guidelines for BACI designs [42]. The differences between control and treatment sites were computed and compared before and after treatment by Mann-Whitney test. SPSS v. 15.0 was used for data processing and statistical analyses.

## Results

### Project implementation

**Figure 3:**
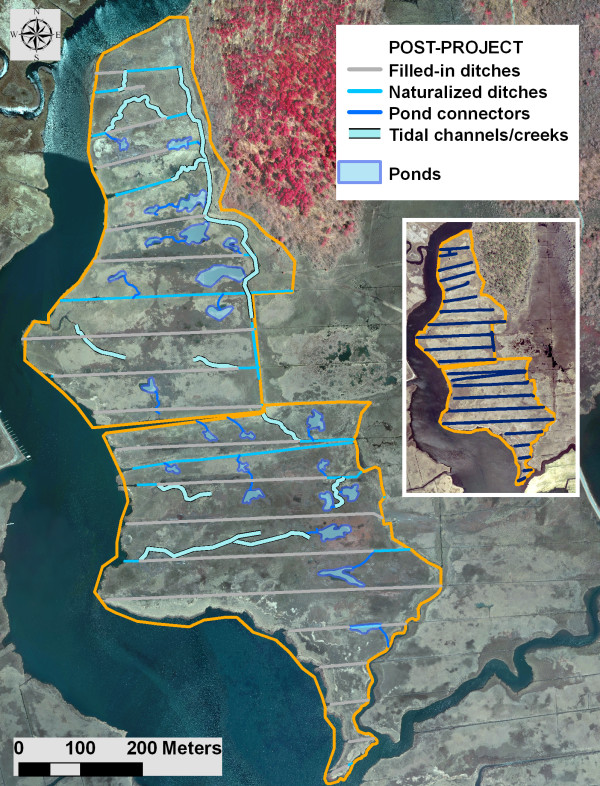
**Salt marsh alterations in Areas 1 and 2 (i.e. treatment) post-project**. Insert shows grid-ditching system (blue lines) pre-project.

Twenty three ponds with the approximate total surface area of 1.1 ha were constructed in the treatment Areas 1 and 2 (Figures 2B and 3). The ponds were constructed with a "teaspoon" profile with at least one deeper sump approximately 0.5–0.75 m deep to serve as a fish refuge. The gradually sloping sides of the ponds were intended to allow fish access into the marsh during flooding, and to serve as foraging habitat for shorebirds. In Area 1, 11 ponds with the size range of approximately 191–1678 sq. meters (mean = 556 sq. meters), and the total area of 0.61 ha were constructed and the material used to completely fill 9 out of 11 existing mosquito grid ditches. The remaining 2 ditches were naturalized and incorporated into a tidal creek system with a new tidal channel reaching into the upland larval habitat and areas heavily infested by *P. australis*. In Area 2, 12 ponds with the size range of approximately 181–887 sq. meters (mean = 432 sq. meters), and the total area of 0.52 ha were constructed and the material used to fill completely or partially 10 out of 11 existing mosquito grid ditches. The ponds constituted about 3% of the total surface area in the treatment marshes. Assuming 1 m as an average width of a filled ditch, the net gain of the open water habitat due to pond construction within the treatment areas was about 0.6.ha or about 1.7% of the total area. The remaining ditch and ditch spurs were naturalized and incorporated into a tidal system following what appeared to be remnants of pre-ditching channels (Figure 3). The pre-existing channel bordering the east side of the area was extended. No alterations were carried out in the control area (Areas 3 and 4), which represented a mix of low and high marsh with significant *P. australis *presence and intact grid ditching system. All four areas pre- and post-treatment are shown in Figures 2 (aerial imagery) and 3 (treatment areas post-project).

### Entomological data

A total of 12,946 samples (dips) were collected in the study area between 2004 and 2008. The sampling effort was fairly uniform (Table 1). Some of the samples were "dry", i.e. no standing water was present at the sampling station (transects), or at the previously identified mosquito larval habitat (targeted), usually during the periods when tidal or rain surface water was completely drained from the salt marsh. Approximately 49% of the transect samples were dry, significantly higher than ~13% of the targeted samples (Chi-square test, *X*^2 ^= 1859.9, df = 2, *P *< 0.001; Table 2). Only about 10% of transect samples with water contained mosquito larvae, which was significantly lower than ~27% in targeted sampling (Chi-square test, *X*^2 ^= 211.4, df = 1, *P *< 0.001; Table 3). Overall, only ~7% of all positive dips came from transect sampling.

**Table 1:** Number of samples (dips) taken per area per year.

**Year**	**Area 1**	**Area 2**	**Area 3**	**Area 4**	**Total**
**2004**	658	588	360	418	2024
**2005**	353	458	339	497	1647
**2006**	644	950	729	1022	3345
**2007**	856	823	621	855	3155
**2008**	723	658	677	717	2775

**Total**	3234	3477	2726	3509	12946

**Table 2:** Proportion of samples (dips) containing water in transect versus targeted sampling. N/R – not recorded.

**Samples (Dips)**		**Water**			**Total**
		Yes	No	N/R	
**Transect**	Count	1646	1590	35	3271
	*%*	*50.3*	*48.6*	*1.1*	
**Targeted**	Count	8152	1227	296	9675
	*%*	*84.3*	*12.7*	*3.1*	

**Table 3:** Proportion of positive samples (dips containing mosquito larvae) per samples with water in transect versus targeted sampling.

**Samples (Dips)**		**Larvae**		**Total**
		Yes	No	
**Transect**	Count	172	1474	1646
	*%*	*10.4*	*89.6*	
**Targeted**	Count	2233	5919	8152
	*%*	*27.4*	*72.6*	

Immature stages of 3 mosquito species, *Ae. sollicitans*, *Ae. cantator*, and *Cx. salinarius *were collected during the study period. The presence of *Cx. salinarius *in the upper salt marsh was unexpected; thus, the close association of this species with the salt marsh habitat was investigated and characterized [7]. Although *Ae. sollicitans *was the most commonly found species in all 4 areas throughout the season, *Cx. salinarius *sometimes predominated in Area 3 when more permanent brackish water from rain events accumulated on the marsh during this species' peak season from late July through early September.

### Entomological data evaluation and statistical analysis

**Figure 4:**
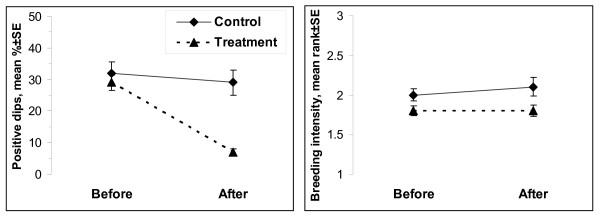
**Weekly mean percent of positive dips ± SE containing larvae and weekly mean breeding intensity, rank ± SE (1 = low, 2 = intermediate, 3 = high; positive dips only) in treatment (Areas 1 and 2) and control (Areas 3 and 4) areas before and after treatment**.

**Figure 5:**
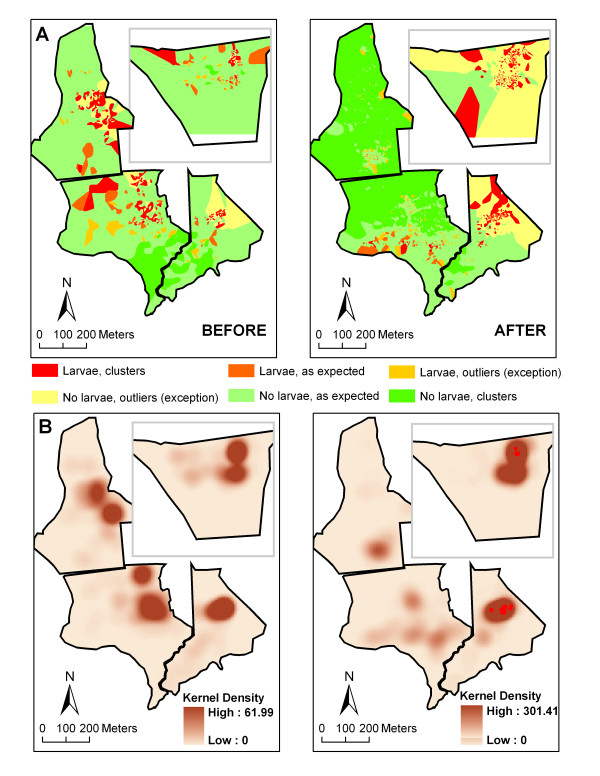
**A) Likelihood of finding larvae (from high to low) before and after the project implementation based on Anselin Local Moran's I**. B) "Hot spots" of elevated breeding intensity (positive dips only) based on Getis-Ord Gi* (Kernel density based on number of positive dips; Red dots = clusters of high values Z > 2.0). Treatment: Areas 1 and 2, Control: Areas 3 and 4.

The proportion of positive dips (i.e. those containing larvae, mean% ± SE) was reduced from 28.8% ± 4.1 to about 7.5% ± 1.1 in the treatment areas (Areas 1 and 2; Figure 4) post-project, while remaining similar (31.9% ± 3.6 versus 29.2% ± 2.5) in the control areas (Areas 3 and 4; Figure 4) post-project. The spatial characterization of the larval production over the entire study area before and after project implementation is shown in Figure 5A. The overall data pattern was highly clustered and auto correlated both before (Global Moran's I = 0.17, Z-score = 29.4, *P *< 0.01) and after (Global Moran's I = 0.20, Z-score = 101.8, *P *< 0.01) the impact. Anselin Local Moran's I identified statistically significant clusters of larval production in all 4 areas prior to the intervention. These clusters were completely eliminated from Area 1 and significantly reduced in Area 2 following the project implementation in these two treatment areas. Conversely, significant clusters of larval production present in the control areas (Areas 3 and 4) pre-project remained in place post-project resulting in highly contrasting pattern between the treatment (absence of larvae clustering) and control (presence of larvae clustering surrounded by negative outliers) areas. Overall, the breeding intensity, i.e. the average number of larvae found in positive dips, remained similar in both treatment and control areas (Figure 4) post-project. However, spatial analysis showed locations of elevated breeding intensity clustered both before and after the intervention (Getis-Ord General G = 0, Z-score = 3.8, *P *< 0.01), but statistically significant clusters were found exclusively in the control area (Areas 3 and 4) following the intervention demarcating focal points of elevated larval production (Figure 5B).

**Figure 6:**
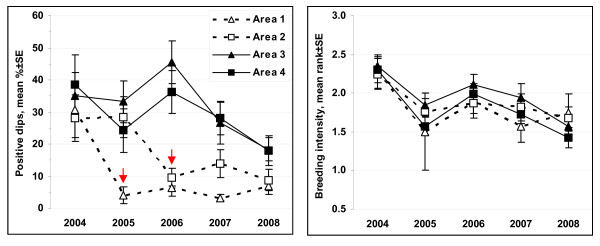
**Weekly mean percent of positive dips ± SE containing larvae and weekly mean rank breeding intensity ± SE (1 = low, 2 = intermediate, 3 = high) in positive dips by in treatment (Areas 1 and 2) and control (Areas 3 and 4) areas by year**. An arrow indicates the first post- treatment year.

**Figure 7:**
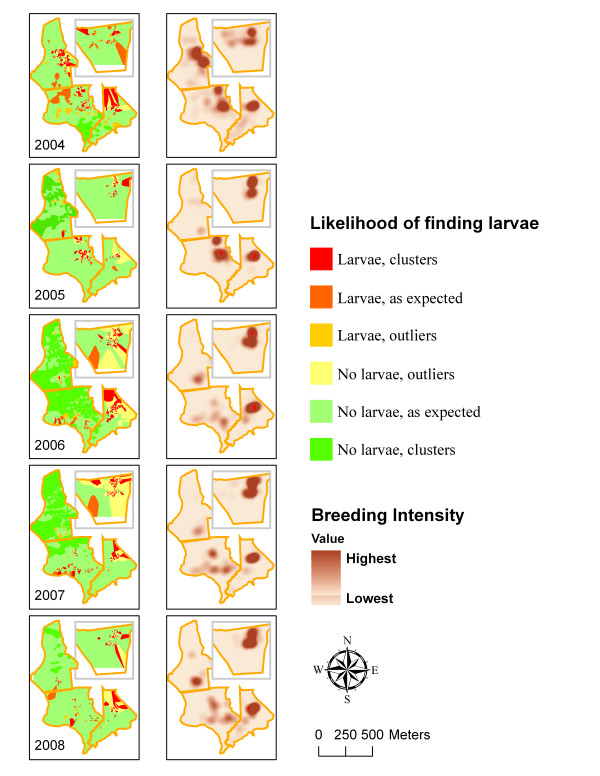
**Left Column: Likelihood of finding larvae (from high to low) by year based on Anselin Local Moran's I**. **Right Column: **"Hot spots" of elevated breeding intensity based on Getis-Ord Gi* (Kernel density based on number of positive dips; ● Red dots = clusters of high values Z > 2.0). Treatment: Areas 1 (2005–08) and 2 (2006–08), Control: Areas 3 and 4.

The steep reduction in the proportion of positive dips occurred immediately after the intervention in both Area 1 (year 2005) and Area 2 (year 2006), whereas no such changes were observed in Areas 3 and 4 (Figure 6). This reduction in larval production remained consistent through 2008. Similarly, the drastic changes in the spatial patterns of larval production within the treatment areas occurred immediately following the intervention and remained consistent through 2008 (Figure 7). Although the overall larval distribution remained highly clustered each year (*P *< 0.01), statistically significant larval clusters in Area 1 were greatly reduced in 2005 (1^st ^post-project season) and eliminated by 2006. Similarly, statistically significant larval clusters in Area 2 were greatly reduced in 2006 (1^st ^post-project season); some residual mosquito breeding occurred on some of the restored marsh surface on top of the filled-in mosquito ditches (see the next section). Unlike the treatment areas, the control areas (Areas 3 and 4) exhibited remarkable consistency, with statistically significant clusters of larvae found in the same geographic locations each year despite discernible inter-annual variability (e.g. "low clustering year" = 2005 and "high clustering year" = 2006). The breeding intensity (based on positive dips only) remained similar in both treatment and control areas through 2008 (Figure 6). Spatial analysis indicated clustering of the locations with elevated breeding intensity in all years (*P *< 0.01) but 2007 (*P *= 0.1). Statistically significant clusters were generated sporadically and occurred in Area 2 (pre-project) and Area 3 in 2005, and were limited to the control areas only post-project (Figure 7).

**Figure 8:**
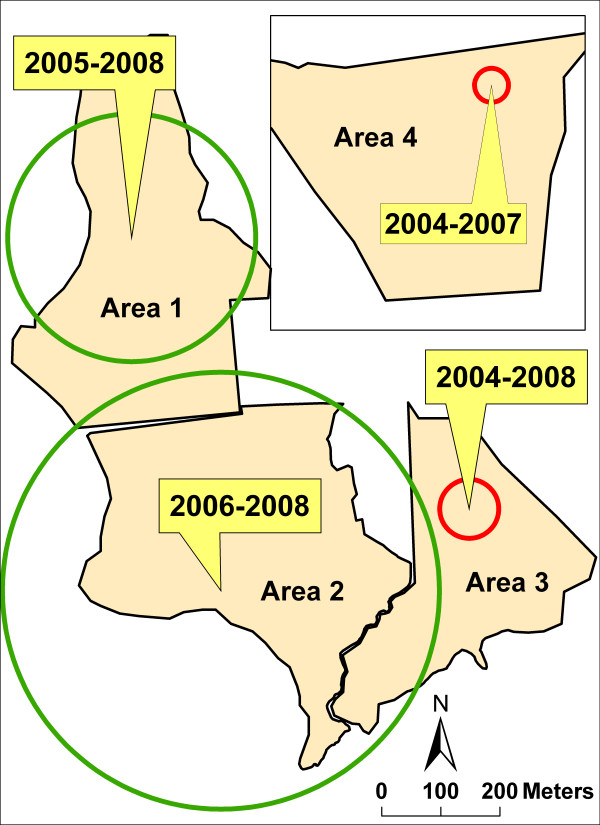
**SaTScan™ space-time cluster analysis of larvae presence or absence**. Geographic extent of statistically significant (*P *< 0.001) clusters of larval presence is indicated by a red circle; geographic extent of statistically significant (*P *< 0.001) clusters of larval absence is indicated by a green circle. Statistically significant time period, years at *P *< 0.001 is shown in yellow boxes. Treatment: Areas 1 (2005–08) and 2 (2006–08), Control: Areas 3 and 4.

To assess statistical significance of patterns of larval production both spatially and temporally, a space-time scan analysis was performed (Figure 8). Most of the treatment areas (Areas 1 and 2) were contained within 2 statistically significant (*P *< 0.001) clusters of low, i.e. "no larvae found" values. These low clusters became significant immediately following the intervention (2005 for Area 1 and 2006 for Area 2) and remained so until 2008. The control areas (Areas 3 and 4) contained 2 statistically significant (*P *< 0.001) clusters of high, i.e. "larvae present" values. Area 3's high cluster was present throughout the study period (2004–08); Area 4's high cluster was not statistically significant in 2008.

### Characterization of the post-project residual larval habitat in the treatment areas

**Figure 9:**
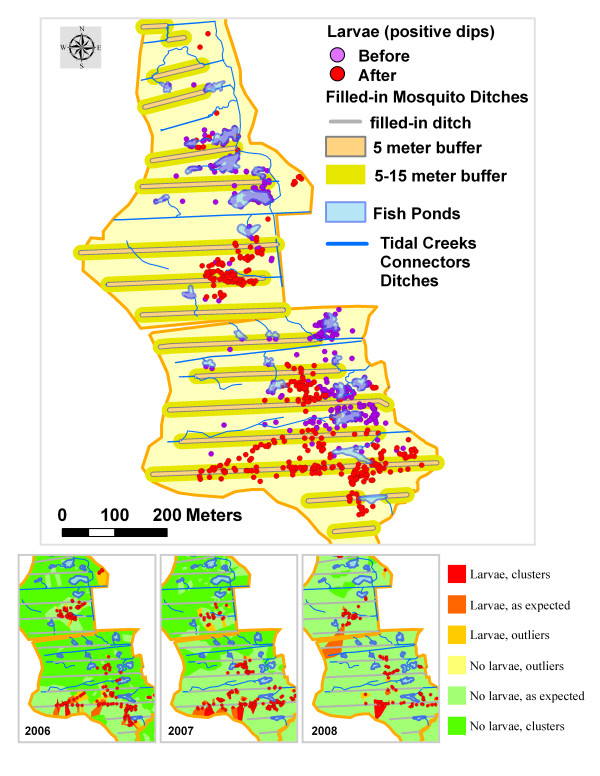
**Post-project problem areas**. Likelihood of finding larvae (from high to low) by year (2006–2008) based on Anselin Local Moran's I is indicated by the color ramp.

Despite significant reduction in the frequency of positive dips within the treatment areas (Areas 1 and 2), some residual breeding continued post-project. Field personnel noted that a considerable proportion of post-project larvae collections were made from the filled-in mosquito ditches, especially in Area 2 (Figure 9). In some ditches, the topsoil used as a fill settled down thereby forming a slightly concave surface, which held standing water suitable for mosquito larvae. Statistical analysis confirmed the role of the filled-in ditches as larval habitat. Pre-intervention, before the ditches were filled to restore the marsh surface in the treatment areas, only a minute proportion (~2%) of positive dips were found in close vicinity (i.e. within 5 meters) of these ditches compared to about one-quarter (~27%) of the total after being filled in (Table 4). Only ~17% of the positive samples were collected within 15 meters from these ditches pre-project compared to ~60% post-project. These differences were statistically significant (Chi-square test, *X*^2 ^= 182.6, df = 2, *P *< 0.001) and corroborated the hypothesis that filled in ditches represented the main residual larval habitat for mosquitoes post-project. However, only a small proportion of the restored marsh surface supported larval habitat. Mosquito larvae were mostly found near 3 out of 19 filled-in ditches and limited to ~835 linear meters (21.6%) out of ~3,867 total filled-in linear meters.

**Table 4:** Number of positive dips as a function of distance to filled in mosquito ditches before and after the intervention.

**Treatment**		**Distance to filled ditch, m**		
		5	5–15	> 15
**Before**	Count	10	66	371
	*%*	*2.2*	*14.8*	*83.0*
	Std. Residual	**-6.6**	**-3.7**	**5.4**
**After**	Count	107	131	161
	*%*	*26.8*	*32.8*	*40.4*
	Std. Residual	**7.0**	**4.0**	**-5.7**

Standardized residual (significant if > 2.0 or < -2.0, bold letters) is indicated.

Inspection of the post-project maps showing the distribution of larvae in the treatment areas (2006 to 2008) indicated a trend of diminishing residual larval habitat. This was also supported by the decreased likelihood of clustering in Area 2, where most of the residual breeding occurred (Figure 9). On the ground, many sections of the filled-in ditches that had been bare and concave with ample larval habitat in 2006–07 were rapidly re-vegetating and becoming level with the marsh surface in 2008. Based on these observations, the process of reduction in the mosquito habitat on or near filled-in ditches is expected to continue in the future.

In addition to the filled-in ditches, two problems of a lesser magnitude contributing to residual mosquito breeding were identified: a) insufficient connectivity with some of the newly created ponds and tidal channels, potentially leading to limited access of the affected areas by larvivorous killifish, and b) increased water accumulation due to a plugged ditch. However, these problems were minor and could be easily corrected without significant impact on the salt marsh surface by either creating shallow radials or removing the plug.

### Mosquito abatement

The mean number of larvicide applications per month during the mosquito season (May–September) for each area is shown in Table 5 for 4 years preceding the project (2001–04) and 4 years post-project (2005–08). The pre-project period was extended by 3 years for this analysis to increase the statistical power and to minimize random trends.

**Table 5:** Mean number of larvicide applications per month (May–September) in 2001–2008. Post-project values (treatment areas) are indicated in bold print.

**Year**	**2001**	**2002**	**2003**	**2004**	**2005**	**2006**	**2007**	**2008**
**Area1**	2.4	1.6	2.0	1.8	**0.2**	**0.4**	**0.2**	**0.6**
**Area2**	2.4	1.6	2.0	1.8	1.0	**0.8**	**0.8**	**0.6**
**Area3**	2.4	1.6	2.0	1.8	1.0	2.6	1.4	1.6
**Area4**	2.8	1.4	2.8	1.8	1.4	2.6	2.2	1.6

**Figure 10:**
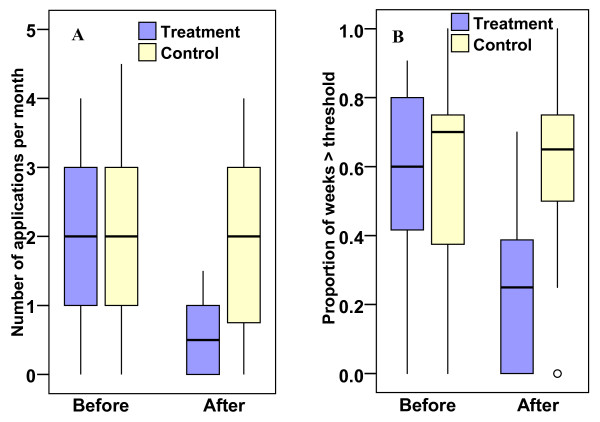
**A) Number of monthly larvicide applications before and after treatment**. B) Proportion of weeks per month when the treatment threshold (> 0.2 larvae/dip) was reached. Minimum and maximum (bar), interquantile range (box), median (horizontal line), and outliers (circle) are shown.

**Figure 11:**
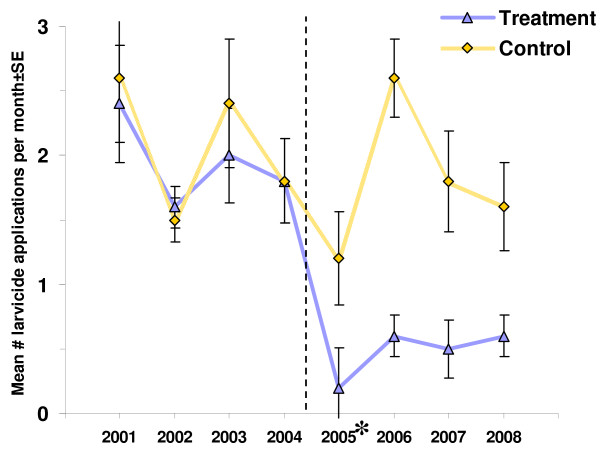
**Mean number of larvicide applications ± SE per month (May–September) in treatment (Areas 1 and 2) and control (Areas 3 and 4) areas**. Before and after is indicated by a dotted line. Dotted bars show the differences between treatment and control areas. *Area 1 was the only treatment area in 2005.

The number of larvicide applications per month (mean ± SE) in the treatment areas was reduced by approximately 74% from 1.95 ± 0.20 pre-treatment to 0.51 ± 0.10 post-treatment. The number of applications (mean ± SE) in the control areas remained similar, 2.08 ± 0.20 pre-treatment to 1.80 ± 0.19 post-treatment. The treatment-control difference was significantly higher post-project (Mann-Whitney U = 61.0, *P *< 0.001) indicating a statistically significant reduction in the number of larvicide applications (Figures 10A and 11). The treatment and control areas pre-project similarities and post-project differences were consistent throughout the study period (Figure 11).

To determine whether the observed reduction in the average number of monthly larvicide applications was due to reduced frequency of positive dips as suggested by the spatial data analysis, the average proportion of weeks per month when the larviciding threshold (> 0.2 larvae/dip) had been reached was computed for each area and compared before (2004–05) and after (2005–08) treatment (Figure 10B). To increase the sample size, Area 2 was compared to Areas 3 and 4 to provide pre-treatment data for 2005, while Area 1 was also compared to the two control areas to provide post-treatment data for the same year. The average proportion of weeks (mean ± SE) above the threshold level for Areas 1 and 2 decreased by approximately 44% from 0.57 ± 0.1 pre-treatment to 0.25 ± 0.05 post-treatment, while that for the control areas remained similar, 0.57 ± 0.1 pre-treatment and 0.60 ± 0.05 post-treatment. The difference in the proportion of weeks between the treatment and the control areas was significantly higher post-treatment (Mann-Whitney U = 21.5, *P *= 0.001) suggesting a statistically significant reduction in the number of weeks when larvicide applications were justified in Areas 1 and 2 following treatment.

## Discussion

This study's goals, design, and analysis differed significantly from the previous investigations on Open Marsh Water Management (OMWM). The goals of the Wertheim Integrated Marsh Management (IMM) project were not limited to achieving reduction in mosquito production (the OMWM component), but also concurrently included restoring the marsh surface by eliminating grid-ditching, and controlling the invasive species *P. australis*. To better assess the impact of these techniques on the marsh flora and fauna, a quasi-experimental before-after-control-impact (BACI) study design with 2 pairs of impact and control sites was utilized. A BACI approach was selected because it offered the closest approximation of a field study to a full experimental design to detect and evaluate the impact (i.e. treatment) effects [35,42]. For mosquito production, geostatistical analysis of the spatial pattern of larval distribution on the marsh surface was used as an alternative to conventional statistical methods to improve the validity of the statistical analysis, to better assess the project effectiveness, and to fully understand the underlying causes of some of the challenges encountered during the study.

Initially, we planned to use random sampling at transect locations to evaluate changes in mosquito production by conventional statistical analysis [34] supplemented by geostatistical analysis of targeted sampling traditionally employed by mosquito control districts. However, about one-half of transect samples were dry (= "no data"), significantly higher than about 13% of those for targeted samples. In addition, only about 9 transect samples with larvae compared to about 112 targeted samples were collected on average per Area each year. Due to high degree of spatial dependency or autocorrelation, the amount of information in the few positive transect samples was further reduced leading to smaller effective sample size, underestimated variance, and increased type I error [43]. Thus, a statistical analysis based exclusively on the transect samples would result in a very low statistical power from a purely technical perspective and questionable biological significance. Numerically superior targeted samples, on the other hand, violated two classical inference assumptions, namely independency between samples (similarly to transect samples) and random selection of the sampling locations. To circumvent these two issues commonly encountered in vector control practice, geostatistical approach was used instead. Presence of spatial dependency is one of the central assumptions in geostatistics, and its magnitude is an important parameter for assessing the spatial patterns such as larval clustering. Although probability sampling is required for an unbiased estimate of population parameters in conventional statistics, geostatistical model-based approach does not require random selection of sampling location [43]. In addition, the representativeness of targeted sampling design used in this study was enhanced by the spatial scope seeking to encompass the entire population and by replication in time over a 5-year study period.

Commonly, quantitative assessment of larval populations relies on number of mosquito larvae per dip. However, this number is highly variable and dependent on many factors unrelated to true mosquito density. For example, mean number of larvae per dip varied significantly both among different operators and between repeated samples taken by the same operator from the same source [44]. Dipper samples could not differentiate population densities below ~280/m^2^, and more than 6,000 samples were required to estimate the population parameters with *α *= 0.05 and *β *= 0.1 [45]. Other factors more specific to the salt marsh mosquitoes may include the size of the pools, presence of larval aggregates, and time of the day, among other factors. Thus, Service's [46] extensive review of entomological literature concluded that larvae per dip could not serve as a true estimate of the larval population. Accordingly, we used presence/absence of mosquito larvae as the main mosquito population parameter in this study. From an operational perspective, the location and the geographic extent of larvae producing areas (i.e. "hotspots") are more important for implementing targeted mosquito abatement program. Large areas of salt marsh mosquito larval habitat can be rapidly characterized using presence/absence data entered into a handheld GPS unit while minimizing technical errors and increasing effective utilization of field personnel. Given similar breeding intensity (i.e. average number of larvae in positive dips) between the treatment and the control areas, frequency of positive samples was also directly proportional to the mean number of larvae per dip in this study.

The OMWM concept was originally developed to provide effective long term control of mosquito larvae by source reduction and biological control. Field data on OMWM projects collected over a 40-year period have been largely supportive of this statement. The magnitude of the reduction in mosquito production generally ranged from 85% to complete elimination [29,33]. For example, Ferrigno [47] reported a reduction from 3.7 × 10^6 ^larvae/acre pre-OMWM to almost zero post-OMWM in the upper marsh *S. patens *treatment areas, while 1.5 × 10^6 ^larvae/acre were detected on average in the control areas. Similarly, a 99% difference of 3.3 versus 0.02 larvae/dip was found between ditched marsh control and OMWM sites in Massachusetts [48]. Meredith and Lesser [29] found 92% reduction in larval densities and 78% reduction of finding mosquito larvae (i.e. frequency) on average summarizing the results of a 28-year OMWM implementation in Delaware. James-Pirri et al., [34] also observed reduction in both the proportion of time the mosquitoes were present and the larval density, although these trends were somewhat obscured by the parameter variability at the control sites. In our study, the frequency of finding larvae in the treatment areas post-project was reduced by ~70% on average, while remaining essentially identical in the control areas pre and post-project. Although the magnitude of this change was somewhat lower than that typically reported in the literature for an OMWM project, this reduction led to marked differences in spatial patterns of larval distribution on the ground. Statistically significant clusters of larvae were no longer present in the treatment areas, but consistently remained in place in the control areas post-project. Moreover, most residual breeding in the treatment areas post-project did not overlap with the pre-project larval habitat, but occupied a new niche atop or near the filled-in ditches created to restore the marsh surface. This finding highlights the potential negative consequences of marsh restoration for mosquito production if new larval habitat is generated during the process. Some of the important mosquito vectors in our area such as *Cx. salinarius*, can successfully utilize both heavily disturbed and relatively pristine marshes [7], which may result in an increased mosquito production from restored areas under favorable environmental conditions. However, even with almost complete elimination of the grid ditching system, only about 21% of the filled-in locations supported new larval habitat suggesting that the majority of the grid ditches contributed very little or none to mosquito larval habitat. For that reason, restoring the marsh surface by removing grid ditching is conceivable if proper surveillance measures to identify problem areas are implemented. GPS based monitoring and GIS/geostatistical techniques such as those described in this article represent crucial components of any surveillance program if a comprehensive project evaluation is required.

Despite the residual mosquito larval habitat in the treatment areas, the OMWM ultimate goal of significantly reducing larviciding while providing sufficient mosquito control was accomplished. The number of larvicide applications was lowered by about 74% in the treatment areas following treatment. This was due to two factors that directly affected the pesticide application criteria: the larviciding threshold and the spatial extent of the mosquito breeding areas on the marsh surface. The larviciding threshold of 0.2 larvae per dip was reached less frequently in the treatment areas by approximately a factor of 2 following treatment due to fewer positive samples containing larvae. The number of larvae per positive dip (i.e. breeding intensity), however, remained similar between treatment and control areas. This observation may be attributed to highly efficient predation of mosquito larvae by killifish in the accessible areas within Areas 1 and 2, whereas locations in the same treatment areas not easily accessible by larvivorous killifish and containing larval habitat (such as the surface of the newly filled-in ditches) continued to support mosquito breeding at the similar intensity to that of pre-project. Changes in the spatial distribution of the mosquito larvae, with reduced extent and loss of clustering in treatment areas also contributed to fewer larvicide applications compared to those in the control areas (Figures 5 and 7). Although 74% reduction in number of larvicide applications is slightly lower than 90–100% reported by other investigators [29,32,49], this difference may be attributed to the expanded scope of this project (i.e. marsh restoration discussed above), lower larviciding thresholds, and more rigorous monitoring procedures.

Continuation of larviciding activities throughout the study period illustrates the difficulties in conducting large scale experiments in natural settings. As was noted previously, pesticide application may confound the results on mosquito production making their interpretation more difficult [34]. To avoid potential bias, Wertheim IMM adopted a set of criteria for larviciding triggers, which were uniformly applied to both treatment and control areas. Using these criteria, the treatment areas consistently received significantly fewer larvicide applications during the post-treatment period (Table 5). In this case, the confounding effect of larviciding would be expected to lessen the differences in mosquito production between treatment and control areas thus leading to a decrease in the before-after effect. However, the differences attributable to OMWM were not only detectable, but statistically significant. Control areas supported higher mosquito production despite retaining intact grid ditching and being subjected to 3–4 times more larvicide applications than did the treatment areas post-project. Thus, the OMWM component in this IMM project demonstrated its potential to largely replace chemical control and marsh-wide parallel grid ditching for effective larval mosquito control.

## Conclusion

This study investigated the effectiveness of Open Marsh Water Management (OMWM) for mosquito vector control when combined with salt marsh restoration and invasive plant species control. Significant reduction was achieved in the frequency of finding larvae on the marsh surface leading to loss of spatial larval clusters or "hotspots" in the areas under OMWM. In turn, these changes resulted in a significant decline of the number of larvicide applications in those areas. Random transect sampling was inadequate to assess the mosquito larval population due to a large proportion of dry "no data" sampling points. More informative targeted sampling necessitated extensive application of GPS and GIS tools to collect and analyze the data using geostatistical methods as an alternative or a supplement to the conventional inference statistics. Geographic analysis was also instrumental in identifying the residual post-OMWM larval habitat, which was largely confined to some of the restored marsh surface, albeit only in a relatively small fraction of the total. Overall, geospatial analysis proved to be a highly useful tool in evaluating the project and more completely understanding how the marsh alterations impacted mosquito larval habitats.

Although mosquito breeding was greatly reduced but not eliminated in OMWM marshes, this technique demonstrated considerable potential for effective mosquito control, which is also compatible with other natural resource management goals such as restoration, wildlife habitat enhancement, and invasive species abatement. No further interventions have been carried out in the treatment areas up to date. In the future, limited scope refinements to reduce or eliminated the remaining larval mosquito habitat may be considered based on geospatial surveillance results.

## Abbreviations

BACI: Before After Control Impact; GIS: Geographic Information Systems; GPS: Geographic Positioning System; IMM: Integrated Marsh Management; OMWM: Open Marsh Water Management; USFWS: United States Fish and Wildlife Service

## Competing interests

The authors declare that they have no competing interests.

## Authors' contributions

IR carried out entomological field work, generated maps, performed the statistical analysis, and drafted the manuscript. TI made significant contributions to the study's conception, design, and implementation, coordinated GIS data processing and map production, and created custom software and geodatabases for the study. MED coordinated and carried out the field data collection. DVN is the principal investigator who conceived of and designed the study, directed the study implementation, and supervised data collection. All authors participated in the result interpretation and manuscript revision.

## Note

The full report on Wertheim National Wildlife Refuge Integrated Marsh Management project is available at:



This website contains links to the comprehensive 2003–2007 report and the data sets of various parameters monitored during the project including mosquito larval surveillance.
